# Correlates of early neonatal feeding practice in Dabat HDSS site, northwest Ethiopia

**DOI:** 10.1186/s13006-017-0116-y

**Published:** 2017-06-06

**Authors:** Terefe Derso, Gashaw Andargie Biks, Amare Tariku, Nigusie Birhan Tebeje, Zemichael Gizaw, Kindie Fentahun Muchie, Alemayehu Shimeka, Yigzaw Kebede, Solomon Mekonnen Abebe, Mezgebu Yitayal, Tadesse Awoke Ayele, Mamo Wubeshet, Temesgen Azmeraw, Melkamu Birku, Abel Fekadu, Geta Asrade, Abebaw Gebeyehu, Adino Tesfahun, Kassahun Alemu

**Affiliations:** 1 Department of Human Nutrition, Institute of Public Health, College of Medicine and Health Sciences, University of Gondar, Gondar, Ethiopia; 2 Department of Health Service Management and Heath Economics, Institute of Public Health, College of Medicine and Health Sciences, University of Gondar, Gondar, Ethiopia; 3 Department of Nursing, College of Medicine and Health Sciences, University of Gondar, Gondar, Ethiopia; 4 Department of Environmental and Occupational Health and Safety, Institute of Public Health, College of Medicine and Health Sciences, University of Gondar, Gondar, Ethiopia; 5 Department of Epidemiology and Biostatics, Institute of Public Health, College of Medicine and Health Sciences, University of Gondar, Gondar, Ethiopia; 6 Dabat Research Centre Health and Demographic Surveillance System, Institute of Public Health, College of Medicine and Health Science, University of Gondar, Gondar, Ethiopia; 7 Department of Reproductive and Child Health, Institute of Public Health, College of Medicine and Health Sciences, University of Gondar, Gondar, Ethiopia

**Keywords:** Early initiation of breastfeeding, Dabat, Northwest, Ethiopia

## Abstract

**Background:**

Delaying the start of breastfeeding and giving prelacteal feeding leads to a significant increase in neonatal and infant deaths, particularly in a resource limited countries, like Ethiopia. Therefore, this study aimed to assess early neonatal feeding practice and its determinants in Dabat HDSS site, northwest Ethiopia.

**Methods:**

The census for the reconciliation of the surveillance of the Dabat Health and Demographic Surveillance System (HDSS) site was conducted from October to December 2014. Data were entered into the Household Registration System (HRS) version 2.1 and analyzed using Stata version 14. A total of 6,761 mother-child pairs were included in the study. Sociodemographic factors, maternal health care and early neonatal feeding practices (early initiation of breastfeeding and prelacteal feeding) were collected by interviewing the mothers. The prevalence of early/timely initiation of breastfeeding was computed as the ratio of children put to the breast within one hour of delivery to the total number of children. Prelacteal feeding was defined as giving anything to drink other than breast milk in the first three days following birth. Binary logistic regression models were used to identify variables which were associated with the dependent variable. A multivariable logistic regression analysis was carried out to identify factors associated with early initiation of breastfeeding.

**Results:**

The prevalence of early initiation of breastfeeding was 43.9% (95% CI, 41.6, 46.2). More than half (56%) of the mothers gave prelacteal feeds. An urban residence (Adjusted Odds Ratio [AOR] 1.47, 95% Confidence Interval [CI] 1.25. 1.73) and antenatal care (AOR 1.41, 95% CI 1.24, 1.59) were correlated with early initiation of breastfeeding. Similarly, increased odds of timely initiation of breastfeeding were observed among mothers who didn’t give prelacteal feeds (AOR 5.72; 95% CI, 5.12, 6.40).

**Conclusion:**

Delayed initiation of breastfeeding and prelacteal feeding still remain public health concerns in this community. The promotion of improved infant and young child feeding (IYCF) practices and the utilization of antenatal care services should be intensified.

## Background

Breastfeeding is one of the most effective ways of ensuring child health and survival [1]. The World Health Organization (WHO) recommends early initiation of breastfeeding and colostrum is produced with its well-known benefits [1, 2]. Breastfeeding within one hour of birth has important benefits both infants and the mothers. It enhances the child’s cognitive development and reduces its risk of developing many infectious. Early breastfeeding also benefits the biological and emotional health of the newborn, helps prevent maternal death by reducing maternal blood loss, and creates a strong and healthy relationship between mother and child [2–6].

Globally, about four million babies die annually in the neonatal period (first four weeks of life), in which almost all (99%) of these deaths are reported to occur in low and middle income countries [7, 8]. In Ethiopia, neonatal and infant mortality rate was 31 and 59 deaths per 1,000 live births, respectively [9]. Suboptimal breastfeeding practices alone, including delay of initiation of breastfeeding and prelacteal feeding cause up to 10% of the global disease burden, and over a million deaths each year in children [10–12]. Thus, endorsement of early initiation of breastfeeding (EIB) is a cost effective intervention and may alone reduce 1.45 million or 22% of all neonatal deaths in developing countries [11, 13]. Furthermore, infants who initiate breastfeeding within one hour of life are more likely to avoid prelacteal feeds and have less chance of being exposed to pathogenic microorganisms [14]. However, in most regions of the world, less than a half of all newborns are put to the breast within one hour in South Asia (42%), West and Central Africa (35%), and sub-Sahara Africa (45%) [15].

Although the Ethiopian Government has developed infant and young child feeding guidelines and a National Nutrition Programme to improve nutrition through realizing and promoting the importance of breastfeeding [16, 17], a sizeable (48% and 27%, respectively) proportion of children were not put to the breast within the first hour of life and were given prelacteal feeds [9].

Sociodemographic and health service related factors, as well as traditional beliefs are the major reasons for poor breastfeeding practices in Ethiopia [9, 18, 19]. Various studies elicited the predictors of the early initiation of breastfeeding, according to which, rural residence [20], being a housewife [21], possession of radio receiver/TV set [21], two or above parity [20], being male infants and mothers in the upper wealth quintiles [22, 23] are associated with increased odds of the early initiation of breastfeeding. On the other hand, illiteracy [24, 25] and lack of antenatal care [26] are correlated with delayed initiation of breastfeeding. Not giving prelacteal feeding has been shown to be associated with the early initiation of breastfeeding [20, 27]. Also, a misconception about the importance of the first breast milk and that giving colostrum caused abdominal cramp, was dirty and the milk was not produce immediately, are the other influencing factor [28].

So far little attention has been given to early neonatal feeding practice by healthcare practitioners and policy-makers [29]. Studies showing the practice and determinants of early neonatal feeding will be of vital importance to address barriers to appropriate early neonatal feeding. Providing evidence on early neonatal breastfeeding practice and its determinant will have a crucial role in improving early neonatal feeding practices. Studies conducted in Ethiopia concerning early neonatal feeding are limited to rural areas and no study has been conducted to address the early neonatal feeding practice in the current study area. Therefore, this study aimed to assess early neonatal feeding and its determinants among mothers in the predominantly rural population of northwest Ethiopia.

## Methods

### Study setting and design

The census for reconciliation for the surveillance of the Dabat Health and Demographic Surveillance System (HDSS) site were conducted from October to December, 2014. The site has been collecting information on vital events, such as birth, death, migration, marital status, housing conditions, pregnancy observation, and pregnancy outcome every six months since Nov.1996. The HDSS covers thirteen randomly selected kebeles (four urban and nine rural) in different ecological zones (high land, middle land, and low land), and has 67,385 inhabitants. For their livelihood the residents largely depend on subsistence farming. The district has four health centers and twenty-nine health posts.

### Study participants

All mothers with children under five years of age and lived in study area for at least six months were included in the study. A total of 6,761 under-five years of age children fulfilling the eligibility criteria were included.

### Data collection tools and procedure

A structured interviewer-administered questionnaire was used to collect data. To maintain consistency, the questionnaire was first translated from English to Amharic (the native language of the study area) and was retranslated to English. Five days’ training regarding the objective of the study, confidentiality of information, and techniques of conducting interview was given to the data collectors and supervisors. A total of 30 data collectors and 11 supervisors were involved in the study.

### Study variables

The outcome variable, early/timely initiation of breastfeeding was determined by asking mothers to provide information regarding the time at which their children were put to the breast after birth. The prevalence of early initiation of breastfeeding was computed as the ratio of children put to the breast within one hour of birth to the total number of children. Prelacteal feeding was defined as giving anything to drink other than breast milk in the first three days following delivery [30]. A mother was asked a key question to ascertain prelacteal feeding practice, ‘within the first three days of delivery, did you give any food or drink other than breast milk to the child?’ Socio-economic status was computed from wealth index information on household assets. Wealth index was ranked and divided into low, middle, and better income tertiles by performing a Principal Component Analysis (PCA).

### Data processing and analysis

Data were entered into Household Registration System (HRS) version 2.1 and analyzed using Stata version 14 statistical package. Frequencies and cross tabulations were used to summarize descriptive statistics; tables and graphs were used for data presentation. Binary logistic regression models were used to identify variables which have associations with the dependent variable. Variables found to have *p*-value up to 0.2 in the bivariable analysis, entered into the multivariable logistic regressions for controlling the possible effects of confounders. Finally, variables which had significant association were identified on the basis of Odds Ratio (OR), with a 95% Confidence Interval.

## Results

### Sociodemographic characteristics

A total of 6,761 mother-child pairs were included in the study. More than three-quarters (77.3%) of the mothers were rural residents. Most of the mothers (87 and 97.8%, respectively) were currently married and reported their religion as Orthodox Christian. Nearly two-thirds (64.8%) of the mothers had no formal education (Table 1).

**Table 1:** Sociodemographic characteristics of study participants in the rural population of northwest Ethiopia, 2014

Characteristics	Frequency	Percent
Age of mothers (in years) (*n* = 6761)		
16–18	283	4.2
19–36	4,654	68.8
37–54	1,824	27
Marital status (*n* = 6761)		
Currently married	5,883	87
Single	113	1.7
Others^a^	765	11.3
Residence (*n* = 6761)		
Rural	5,226	77.3
Urban	1,535	22.7
Religion (*n* = 6761)		
Orthodox Christian	6,625	97.8
Others^b^	136	2
Maternal educational status (*n* = 6761)		
No formal education	4,374	64.7
First cycle (1–4 grade)	935	13.8
Second cycle (5–8 grade)	603	8.9
Secondary school (9–12 grade)	849	12.6
Wealth index/status (*n* = 6554)		
Low income	1,315	20.1
Middle income	2,791	42.6
Better income	2,448	37.3

^a^separated, divorced and widowed ^b^ Muslim and protestant

N.B. The total number of respondents for each variable is not equal because of missing values.

### Healthcare related characteristics of the mothers

About 64.4% of the mothers had an attended antenatal care (ANC). However, a vast majority (92.1%) of mothers consumed alcohol sometimes. Two-thirds (68.6%) of the mothers didn’t use family planning at the time of the study. Few (1.8%) mothers reported suffering from chronic diseases (Table 2).

**Table 2:** Maternal health care related characteristics in the rural population of northwest Ethiopia, 2014

Variables	Frequency	Percent
Antenatal care (*n* = 6389)		
Yes	4,355	64.4
No	2,034	35.6
Before pregnancy use of family planning (*n* = 6761)		
Short term family planning methods	3,367	49.8
Long term family planning methods	194	3.4
No	3,200	46.8
Current use of family planning (*n* = 6389)		
Yes	2,005	31.4
No	4,384	68.6
Alcohol consumption (*n* = 6761)		
Never	406	6
Sometimes	6,225	92.1
Often	130	1.9
Chronic disease^a^ (*n* = 6761)		
Yes^a^	123	1.8
No	6,638	98.2

^a^Diabetes mellitus, Hypertension, Asthma, Heart diseases and Cancer

### Early neonatal feeding practices

The prevalence of early initiation of breastfeeding was 43.9% (95% CI, 41.6, 46.2). Of the total number of mothers, more than half (56%) (95% CI, 53.7, 58.1) gave their neonate something other than breastmilk to drink in the first three days. The most common prelacteal foods given was raw butter (49.1%) (Table 3).

**Table 3:** Early neonatal feeding practice of mothers in the rural northwest Ethiopia, 2014

Variables	Frequency	Percent
Early initiation of breastfeeding (*n* = 6761)		
Yes (within one hour)	2,969	43.9
No (after 1 hour)	3,792	56.1
Time of initiation of breastfeeding (*n* = 6761)		
Within one hour (immediately)	2,969	43.9
1–24 hours	2,190	32.4
After 24 hours	1,602	23.7
Prelacteal feeding (*n* = 6761)		
Yes	3,791	56
No	2,970	44
Types of prelacteal feeds (*n* = 3,791)		
Cow milk	57	1.5
Raw butter	1,861	49.1
Tea	36	0.9
Sugar in water	624	16.5
Pure water	668	17.6
Others^a^	545	14.5

^a^Fenugreek, Ersho and formula milk; Ersho is a traditional baking soda prepared by incubating the flour and double distilled water

### Factors associated with early initiation of breastfeeding

In the bivariate analysis, age, residence, education, marital status, antenatal care and prelacteal feeding were found with a *p*-value of less than 0.2. However, the multivariable analysis revealed that the independent predictors of early initiation of breastfeeding were residence, antenatal care, and prelacteal feeding. The odds of early/timely/initiation into breastfeeding were higher among mothers living in urban areas (AOR = 1.47, 95% CI, 1.25. 1.73). Likewise, mothers who had received antenatal care were 1.41 times more likely to initiate breastfeeding within one hour of birth (AOR = 1.41, 95% CI, 1.24. 1.59). Finally, an increased odds of timely initiation of breastfeeding were observed among mothers who did not give prelacteal feeds (AOR = 5.72; 95% CI, 5.12, 6.40) (Table 4).

**Table 4:** Factors associated with early initiation of breast feeding in the rural population of northwest Ethiopia, 2014

Variables	Early initiation of breastfeeding		Crude Odds Ratio^@^ (95% CI)	Adjusted Odds Ratio^#^ (95% CI)
	Yes	No		
Maternal education				
No formal education	1,767	2,607	1	1
First cycle	386	549	1.04 (0.89, 1.20)	0.97 (0.82, 1.14)
Second cycle	277	326	1.25 (1.06, 1.49)	0.97 (0.79, 1.19)
Secondary school	539	310	2.56 (2.20, 2.99)	1.09 (0.88, 1.34)
Marital status				
Currently married	2,524	3,359	1	1
Single	45	68	0.88 (0.60, 1.29)	0.74 (0.48, 1.15)
Others	400	365	1.46 (1.25, 1.69)	1.19 (0.99, 1.43)
Residence				
Rural	2,061	3,165	1	1
Urban	908	627	2.22 (1.98, 2.50)	1.47 (1.25. 1.73)*
Antenatal care				
Yes	2,135	2,220	1.96 (1.75, 2.19)	1.41 (1.24, 1.59)*
No	670	1,364	1	1
Age of the mothers				
16–18 years	102	181	1	1
19–36 years	2,071	2,583	1.42 (1.11, 1.83)	1.21 (0.91, 1.61)
37–54 years	796	1,028	1.37 (1.06, 1.78)	1.35 (0.99, 1.83)
Prelacteal feeding				
Yes	938	2,854	1	1
No	1,988	981	6.17 (5.54, 6.86)	5.72 (5.12, 6.40)*

^@^Crude Odds Ratio, considering the effect of only one predictor variable

^*^Significant at *p*-value less than 0.05

^#^Adjusted Odds Ratio, which takes into account the effect due to all the additional variables included in the analysis

## Discussion

In this study, the early initiation of breastfeeding was 43.9%; urban residence, antenatal care, and avoiding prelacteal feeding were identified as independent predictors of the early initiation of breastfeeding.

The prevalence of breastfeeding initiation within one hour of birth was fair when compared to the World Health Organization (WHO) recommendations [31]. This was consistent with studies reported from Northern Ethiopia (41.6%) [32], Western Nepal (42.2%) [33] and Nigeria (45%) [34]. However, the current finding was lower than the national studies conducted in Ethiopia (52%) [9], Tanzania (49%) [35] and Kenya (58%) [36]. The difference might be explained by the depth of the studies and the latter reports were, for example, national studies including large cross-cultural differences in breastfeeding practices across communities. However, the current study was done in predominantly rural areas of northwest Ethiopia. The low educational status, poor nutritional awareness, local beliefs and traditions all contribute to suboptimal breastfeeding practices, and that the delayed initiation of breastfeeding is more common in the predominantly rural areas [9, 28, 37]. Compared to the urban settlements, breastfeeding of children is affected by limited healthcare facilities in rural areas [37].

The current study revealed that more than half (56%) of the neonates were given prelacteal feeding, which was similar with the finding reported from Egypt (57.8%) [38]. This finding was more prevalent than that of the 2011 Ethiopian Demographic and Health Survey (EDHS) report (27%) [9]. This could be related to the low maternal healthcare utilization and poor maternal educational status in the study area and suggests that the healthcare services and education are fertile grounds to prevent suboptimal breastfeeding, such as prelacteal feeding. On the other hand, the result was lower than the report from Vietnam (73.3%) [39] and could be explained due to sociocultural differences.

The result of the multivariable analysis showed that the likelihoods of early initiation of breastfeeding were higher among mothers living in urban areas. This was in agreement with a previous national finding in Vietnam, where breastfeeding initiation within one hour of birth was more common in urban areas than in rural areas [37]. An explanation might be the fact that mothers living in rural areas are more likely to be affected by community beliefs towards in delays in the initiation of breastfeeding practices, and their low access to nutrition education and counseling about infant and young child feeding (IYCF). However, a finding from Saudi Arabia showed that rural mothers were more likely to initiate breastfeeding earlier than urban mothers [40]. This might be explained by the fact that in Saudi Arabia the participants had better education and adequate maternity care provided to both urban and rural areas through a network of primary healthcare centers. This suggests that better education and maternal healthcare accessibility create a perfect opportunity for encouraging appropriate breastfeeding practices.

This finding also confirmed that mothers who had antenatal care were 1.41 times more likely to initiate breastfeeding within one hour of birth, and is similar to another study from Nigeria [26]. It may be attributed to the mother’s opportunity to obtain information about the importance of timely initiation of breastfeeding from antenatal counseling sessions. This suggests the significance of the role of information to empower mothers to resist external interferences and pressures from traditional faiths and misunderstandings that encourage the delay in the initiation of breastfeeding. Currently, the focused antenatal care guideline in Ethiopia recommends intensive heath education about breastfeeding at the time of antenatal care visits for mothers.

Furthermore, increased odds of early initiation of breastfeeding were observed among mothers who didn’t give prelacteal feeding. The finding was supported by evidences from the rural parts of Western Ethiopia [21] and Saudi Arabia [20]. As this is a cross-sectional analysis, it is not clear whether prelacteal feeding is a cause or consequence of timely initiation of breastfeeding but this might be due to prelacteal feeding, which might result in weakening the suckling stimulus of the baby [14]. Since the information was obtained from mothers with children aged to 59 months, recall bias could not be ruled out. Also, hence, this study employed the WHO criteria [30] to estimate early initiation of breastfeeding, in which mothers attempt to initiate breastfeeding, was not adequately captured [40]. Consequently, the prevalence of EIB might be overestimated.

## Conclusion

Delay of initiation of breastfeeding and the practice of prelacteal feeding remains public health concerns in this community. Therefore, promotion of improved infant and young child feeding (IYCF) practices and utilization of antenatal care should be intensified.

## Abbreviations

ANC: Antenatal Care
AOR: Adjusted Odds Ratio
CI: Confidence Interval
COR: Crude Odds Ratio
EIB: Early Initiation of Breastfeeding
IYCF: Infant and Young Child Feeding
PCA: Principal Component Analysis
SD: Standard Deviation
WHO: World Health Organization
